# Wheat-Related Disorders in Children: A 360-Degree View

**DOI:** 10.3390/children11060707

**Published:** 2024-06-07

**Authors:** Michele Fingerle, Sebastiano Salaorni, Angelo Pietrobelli, Giorgio Piacentini, Claudia Banzato, Luca Pecoraro

**Affiliations:** Pediatric Unit, Department of Surgical Sciences, Dentistry, Gynecology and Pediatrics, University of Verona, 37126 Verona, Italy

**Keywords:** wheat-related disorders, celiac disease, IgE-mediated wheat allergy, non-IgE mediated wheat allergy, wheat-related eosinophilic esophagitis, non-celiac gluten sensitivity

## Abstract

Immunological illnesses related to wheat represent an epidemiologically relevant phenomenon at a pediatric age. The term “Wheat-related disorders” involves a spectrum of diseases: celiac disease, IgE-mediated wheat allergy, non-IgE mediated wheat allergy, wheat-related eosinophilic esophagitis, and non-celiac gluten sensitivity. Their pathogenesis is different. At the same time, wheat represents their common point. This article aims to the state-of-the-art and new clinical evidence in pediatric age.

## 1. Introduction

Wheat (*Triticum aestivum*) is the world’s most widely consumed food grain [1]. Most of the world’s production is consumed in bread and baked foods, pasta, bulgur, and couscous. Wheat starch and gluten proteins are also used in food processing. The term “gluten” refers to the water-insoluble fraction composed of approximately equal amounts of gliadin and glutenin [1]. It is present not only in wheat but also in rye, barley, and oats. 

In children, this type of food is often introduced in the first months of life (4–7 months) and plays a predominant role in the diet, serving as the foundation of the Mediterranean food pyramid [2,3].

**Figure 1:**
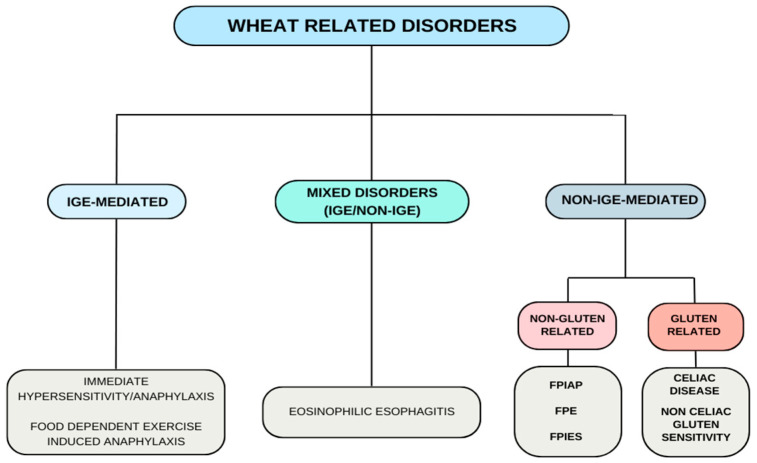
Wheat-related disorders-concept map; FPE: Food Protein Enteropathy; FPIAP: Food Protein-Induced Allergic Proctocolitis; FPIES: Food Protein-Induced Enterocolitis Syndrome.

This review examines wheat-related immune-mediated disorders, including all gluten-related conditions, such as celiac disease, non-coeliac gluten sensitivity, wheat-related eosinophilic esophagitis, and also the different types of food-related allergies (FA) like IgE-FA, mixed IgE/non-IgE-mediated allergies, and non-IgE-GI-FA (Figure 1). 

## 2. Materials and Methods

The electronic databases Medline PubMed Advanced Search Builder, Scopus, Web of Science (from January 1994 to March 2024) were analyzed using the following medical subject headings (MeSH) terms and text words (as well as their combinations and truncated synonyms): “wheat-related disorders”, “wheat allergy”, “celiac disease”, and “non-celiac gluten sensitivity”. The abstracts were reviewed after removal of duplicate articles. The full text of suitable papers was analyzed. The inclusion criteria were studies reporting the results of case reports, case series, case–control studies, cohort studies, synthesized data, reviews, and randomized trials. The search was limited to English language articles. The exclusion criteria included studies published only as abstracts, letters, conference proceedings, discussion papers, animal studies, and editorials. The initial screening of titles was carried out to identify potentially relevant studies, followed by the screening of abstracts and then by full paper reviews. All titles and abstracts were independently evaluated by two reviewers (MF and SS). Quality assessments were conducted by two independent reviewers (MF and SS); at the same time, the supervision was made by the other authors (LP, MB, GP, AP). All data were independently confirmed. After an initial search, an analysis based on the key questions and the inclusion and exclusion criteria narrowed down the results to 71 papers that fitted the inclusion criteria.

## 3. IgE-Mediated Wheat Allergy

Wheat allergy is a prevalent food allergy. Wheat-related food allergies are categorized into IgE-mediated wheat allergy (WA) and IgE-non-mediated WA [4].

### 3.1. Epidemiology

The global prevalence of WA ranges between 0.2 and 1%, with a higher frequency in the pediatric population [5]. It ranks as the third most prevalent allergen in Germany, Japan, and Finland, following milk and eggs [6]. 

### 3.2. Pathogenesis

The contact between the antigen and the immune system in genetically predisposed individuals exposed to specific environmental factors generates an immediate hypersensitivity reaction (type I hypersensitivity). This leads to the production of specific IgE against wheat proteins, which triggers the release of mediators by basophils and mast cells [7]. The potential allergens contained in wheat can be divided into the following four categories: albumins, globulins, glutenins and gliadins [8]. The latter are divided based on electrophoretic characteristics into ∝/β-gliadins, ω-gliadins and γ-gliadins [1]. The glutenins are divided into low and high molecular weight [1]. Among the albumins and globulins, those capable of stimulating an immune response include ∝/β-amylase, proteinases, lipid transfer proteins (LTPs) and puroindolines [1]. 

### 3.3. Clinical Presentation 

Symptoms can appear similar to those of other food allergies, or they may present specific conditions such as wheat-dependent exercise-induced anaphylaxis (WDEIA) [9]. 

Symptoms typically appear within minutes to 1–2 h of consuming wheat. This condition may present with reactions involving the skin, oropharyngeal area, upper and lower respiratory tracts, gastrointestinal tract, and the cardiovascular system. In younger children, gastrointestinal issues such as abdominal pain, diarrhea, and vomiting are more common, whereas teenagers and adults are more likely to experience severe reactions like anaphylaxis [10]. 

### 3.4. Diagnosis

The food challenge continues to be the gold standard for diagnosing WA [9]. The patient is administered whole wheat, beginning with small amounts of wheat-specific protein and gradually increasing the dosage every hour [4]. The emergence of symptoms within two hours of consumption during a challenge test confirms WA diagnosis. Skin prick tests (SPTs) using commercial wheat extract are not very specific. Measuring serum levels of allergen-specific IgE is highly sensitive but features low specificity [4,7]. Molecular allergy tests are also available for Tri a 14, Tri a 19, and gliadins [7].

### 3.5. Treatment and Prognosis

The treatment is represented by a wheat-free diet. Other gluten-containing grains like rye, barley and oats are generally well tolerated by most patients and therefore should not be removed from their diet [9]. According to Czaja-Bulsa et al., the prognosis for wheat allergy is good, with average attainment of tolerance by age 7 and 76% of patients developing tolerance by age 16 [10]. Tolerance correlates with wheat-specific IgE concentration [10]. A recent trial published on NEJM shows that a 16-week course of omalizumab, a monoclonal anti-IgE antibody, can increase the reaction threshold for common food allergens, including wheat proteins [11]. 

### 3.6. Wheat-Dependent Exercise-Induced Anaphylaxis

Wheat components are the most frequently involved in food-dependent exercise-induced anaphilaxis (FDEIA) [12]. FDEIA involves conditions where anaphylaxis is due to the combination of food ingestion and subsequent physical exercise. In some cases, the reaction occurs even if the physical activity precedes exposure to the allergen. The actual prevalence of WDEIA has not yet been well defined. Prevalence studies conducted in Japan by Manabe T. et al. observed a higher frequency of FDEIA in secondary schools compared to children attending primary school (0.018% vs. 0.0047%) [13]. Omega-5 gliadin (tri a 19) is a major allergen found in wheat [13]. The pathogenetic mechanisms of WDEIA are not fully understood. One of the possible mechanisms involved is related to the fact that physical activity increases the release of interleukin-6 (IL-6), which, in cases of concurrent wheat intake, increases the activity of tTG, facilitating the IgE-mediated reaction [14]. There are also cofactors, such as NSAID, that can facilitate the onset of WDEIA through a summation effect. The diagnosis is based on the exclusion of other causes that could explain the allergic reaction and the presence of all of the following points: 1. temporal association between wheat ingestion and physical exercise and onset of signs and symptoms of anaphylaxis; 2. A positive IgE test; 3. allergic reaction does not occur with wheat intake alone or with physical exercise alone [15]. In addition to a skin prick test (SPT) and allergen IgE test, in some cases, it is useful to resort to molecular diagnosis (CRD, component resolved diagnosis) to evaluate IgE sensitization to Tri a 19 [1]. In all doubtful cases, it is possible to perform an oral provocation test in which physical activity (treadmill or cycle ergometer) is required 60 min after the ingestion of the defined amounts of gluten [16]. In some cases where the test has come back negative, it can be repeated with the addition of aspirin [17]. In the differential diagnosis, it is crucial, through a detailed medical history, to clarify whether the reaction is related to the food or to an inhaled antigen. Additionally, flour mites that contaminate grains can erroneously suggest WDEIA in a patient allergic to mites [18]. For the management of WDEIA, it is necessary to avoid the consumption of foods containing wheat within the 4 h prior to physical activity and within the hour following the end of exercise [19]. For younger children, where movement is almost constant, a complete elimination diet may be necessary. Currently, aside from the preventive strategies described above, there are no pharmacological interventions of proven efficacy. 

## 4. Non-IgE Mediated Food Allergies

Non-IgE mediated gastrointestinal food allergies (non-IgE-GI-FAs) encompass conditions like food protein-induced enterocolitis (FPIES), allergic proctocolitis (FPIAP), and enteropathy (FPE), in which food-specific IgE are typically not present.

They are believed to represent a spectrum of diseases where the expression and severity of the condition are influenced by the specific segment of the gastrointestinal tract that is affected. In contrast to IgE-FA, the onset of GI symptoms is often delayed after trigger food exposure and can present a chronic course [20].

### 4.1. Epidemiology

The prevalence and incidence of non-IgE-GI-FA still need to be well established. FPIAP is the most frequent among them, with an estimated prevalence of 2–3% [21]. FPE is considered relatively uncommon and has declined over the last few decades [22]. The identification of FPIES as a rare disease has been questioned by recent reports, revealing a cumulative incidence of 0.3–0.7% in children [23].

### 4.2. Pathogenesis

Non-IgE-mediated gastrointestinal food allergies (non-IgE-GI-FAs) have a complex and not fully understood pathogenesis. They differ from IgE-FA because they involve cellular immunity rather than circulating specific IgE antibodies. Localized IgE responses have also been observed. 

In FPIES, specific T cell responses to food antigens are demonstrated that are associated with an imbalance between TNFα and TGFβ responses. Local T cell infiltration causing intestinal permeability was described as the main causal factor, but recent evidence complicates this view, suggesting a Th2 involvement similar to IgE-FA [24].

In FPE, damage to the jejunal mucosa is linked to food-specific T-cell infiltration, mainly targeting cow’s milk antigens. Cytotoxic CD8+ T cells are implicated, alongside increased intraepithelial γδ-TCR+ cell density [20].

Finally, FPIAP involves eosinophilic infiltration of the rectosigmoid mucosa, but why inflammation localizes to the distal colon remains unknown [24].

### 4.3. Food Triggers

The most commonly reported food allergy in the pediatric population is cow’s milk. Non-IgE-GI-FA account for up to 40–50% of all reactions, and cow’s milk is the most represented food trigger in FPIES, FPE, and FPIAP. However, other foods, such as wheat, egg, corn and soy, have been often implicated [20].

### 4.4. Clinical Features

Acute FPIES possibly affects all parts of the gastrointestinal tract, with its hallmark symptom being severe, uncontrollable vomiting, which can lead to metabolic imbalances and hypovolemic shock. It often occurs 1–4 h after eating. Diarrhea may follow 5–10 h later, though it is less frequent. Infants may show signs of lethargy, pallor, and sometimes hypothermia. Some severe cases present unstable blood pressure. Chronic FPIES, however, manifests as ongoing watery diarrhea, occasional vomiting, abdominal bloating, and poor weight gain. Continued exposure to cow’s milk or soy-based formula typically triggers chronic FPIES, while intermittent exposure to solid foods like rice may cause acute episodes of FPIES. A key aspect of chronic FPIES is the reappearance of acute symptoms after the reintroduction of the trigger food. Although rare, FPIES can occur in exclusively breastfed infants. The age of onset varies, but it typically arises in early infancy [20,25].

FPE primarily impacts the small intestine, leading to lower intestine symptoms. The presenting symptoms develop in infants shortly after the introduction of cow’s milk into the diet, with a clinical presentation closely resembling celiac disease. These include chronic diarrhea, vomiting and malabsorption signs, such as steatorrhea and failure to thrive, which regress after the elimination of cow’s milk from the diet [26]. Patients with FPE can also present a mucosal damage that leads to a secondary carbohydrate intolerance [20].

FPIAP typically affects breastfed infants in their early weeks due to exposure to maternal dietary protein via breast milk. FPIAP symptoms arise from inflammation localized in the distal colon, leading to bloody stools in otherwise well-appearing infants. These infants have bloody, loose stools, sometimes with mucus. Some may also show gagging, food refusal, and irritability, signs of allergic inflammation extending beyond the distal colon. While most common in young infants, FPIAP can also occur in older children [20].

### 4.5. Diagnosis

The diagnosis of non-IgE-GI-FA is based upon the history, a constellation of typical clinical symptoms with clinical improvement following withdrawal of the suspected causal protein, and exclusion of other etiologies [25,26]. In acute FPIES, if necessary, an oral food challenge (OFC) is performed as the gold standard [25,27].

### 4.6. Treatment

The primary management approach for non-IgE-mediated gastrointestinal food allergies involves removing trigger foods from the diet. Two strategies are utilized depending on symptom severity and the quantity of trigger foods. The “bottom-up approach” gradually eliminates causal foods without restricting other tolerated foods. In contrast, the “top-down approach” may be necessary for severe cases, initially avoiding a wide range of foods and reintroducing them sequentially while monitoring symptoms [20,26].

### 4.7. Prognosis

The prognosis for non-IgE mediated gastrointestinal food allergies is largely positive, with most cases being resolved in school-aged children. FPIES and FPE typically regress before 3 to 5 years of age and by 1–2 years of age, respectively. However, in less common instances, these conditions may persist into later childhood. Conversely, FPIAP persistence beyond 1–2 years of age is uncommon [20].

## 5. Celiac Disease (CeD)

### 5.1. Definition, Epidemiology and Pathogenesis

In contrast to the forms described above, celiac disease (CeD) is not due to a sensitization to wheat proteins but to a systemic autoimmune reaction triggered by gluten [28]. It is among the most prevalent chronic conditions, with an overall prevalence of 1%. A recent multicenter study conducted by the Italian Society for Pediatric Gastroenterology, Hepatology and Nutrition (SIGENP) showed that the overall prevalence of celiac disease in school-age Italian children is 1.65% [29]. In fact, over the last 30 years, there has been an increase in the incidence of CeD, as demonstrated by a study conducted in Italy involving more than 4500 children [30]. This is mainly due, on the one hand, to greater awareness of CeD and, on the other hand, to increases in the sensibility of serological testing for CeD.

The development of CeD involves a complex interplay of immunological, environmental and genetic factors. Primarily, the dietary ingestion of gluten causes an inflammatory reaction at the level of the intestinal mucosa, engaging both the adaptive and innate immune systems. A 33-mer peptide from gluten digestion may be the key trigger of this inflammatory response in CeD patients [31]. The human leukocyte antigen (HLA) DR3-DQ2 and DR4-DQ8 are strongly linked to CeD susceptibility. Over 99% of those with CeD have these gene variants, compared to 30–40% of the general population in most countries.

### 5.2. Clinical Presentation

CeD manifests in various ways, ranging from gastrointestinal symptoms and extraintestinal manifestations to asymptomatic cases. Based on clinical features at diagnosis, coeliac disease can be categorized into classical, non-classical, and subclinical forms [32]. The classical form, more common in children under five, presents with chronic diarrhea, failure to thrive, and a distended abdomen due to malabsorption [32]. The most prevalent presentation is the non-classical form; it includes non-specific intestinal complaints, extraintestinal symptoms like iron deficiency and chronic fatigue, and other systemic issues such as nutritional deficiencies, joint pain, alopecia, recurrent stomatitis, and chronic urticaria [32]. In recent decades, there has been a shift from the traditional symptoms of malabsorption to nonclassical, oligosymptomatic, and asymptomatic forms [33]. A retrospective study of over 600 children diagnosed with CeD showed that one-fifth were asymptomatic at presentation [33]. Type 1 diabetes, Hashimoto’s thyroiditis, and autoimmune liver disease are closely associated with CeD [34,35,36]. Moreover, Down’s syndrome, selective IgA deficiency and Turner syndrome are also frequently associated [37,38,39]. 

### 5.3. Diagnosis

Diagnostic criteria for CeD have changed over the past 50 years. Current guidelines from the European Society for Paediatric Gastroenterology, Hepatology, and Nutrition (ESPGHAN) include omitting the small intestinal biopsy if particular laboratory criteria are met [40]. The non-invasive approach is possible when the anti-transglutaminase (tTG) IgA values exceed 10 times the upper limit of normal (ULN) and an endomysial antibodies (EMA-IgA) test is positive in a second blood sample. In children who do not meet these criteria, an endoscopic examination is still required to complete the diagnosis. The Marsh-Oberhuber classification graded the extent of intestinal damage dividing the lesion in infiltrative (type 1), hyperplastic (type 2) and destructive (type 3) cases [41].

All CeD blood or endoscopic tests must be conducted on a gluten-containing diet. 

Potential celiac disease (PCD) is defined as the presence of positive serum antibodies, genetic predisposition, and normal intestinal mucosa (Marsh grade 0–1) [32]. In these cases, a gluten-free diet (GFD) should be considered only if gluten-dependent symptoms are present. Asymptomatic individuals require clinical and laboratory surveillance to monitor for villous atrophy [40]. Pediatric guidelines recommend screening asymptomatic high-risk children, including first-degree relatives of CeD patients, and those with autoimmune conditions, Down’s, Turner and Williams syndrome, selective IgA deficiency, and juvenile chronic arthritis [40].

### 5.4. Treatment

CeD requires a strict, lifelong GFD and, to date, this is the only effective treatment [42,43]. Commercially available gluten-free products must contain less than 20 mg/kg of gluten to be safe [44]. Grains naturally without gluten, such as rice, corn, quinoa, and amaranth, are allowed. While certain oat varieties may contain trace amounts of compounds that can reactivate coeliac disease, this phenomenon is sporadic [44,45]. Non-adherence to a gluten-free diet is more prevalent among males, adolescents, and asymptomatic individuals [46]. Regular small gluten intake may not cause immediate symptoms but can lead to long-term intestinal damage [47]. Future therapeutic approaches for coeliac disease could involve next-generation probiotics targeting specific disease mechanisms or genetically engineered bacteria producing immune-regulatory substances [48]. Most patients see symptom improvement within six months of strict adherence to a gluten-free diet [48]. Monitoring hematological parameters, including iron, folate, and vitamin D levels, is essential for diagnosing and correcting abnormalities. Complete normalization of coeliac disease markers may take over two years, especially in individuals with exceptionally high initial levels of anti-TG2 antibodies [49].

## 6. Non-Celiac Wheat Sensitivity (NCWS)

### 6.1. Definition, Epidemiology and Pathogenesis

Non-celiac gluten sensitivity (NCGS) is a poorly defined condition marked by both intestinal and other symptoms that arise from consuming gluten in individuals who have not been diagnosed with either celiac disease (CeD) or wheat allergy [50]. 

Present knowledge suggests that symptoms of NCGS may be due to components in wheat other than gluten, such as monosaccharides, disaccharides, fermentable oligosaccharides and polyols (FODMAPs), along with amylase-trypsin inhibitors (ATIs) [51]. Due to the lack of specific biomarkers, the diagnosis of NCWS is often self-reported, which complicates the determination of the actual incidence of these disorders. Many patients who experience symptoms from gluten ingestion start a gluten-free diet (GFD) independently of medical advice [50]. In Italy 20% of patients undergoing digestive endoscopy reported symptoms of NCWS [52]. Components like FODMAPs, gluten, and ATIs may trigger responses involving both the immune system and metabolic processes, playing a significant role in NCWS symptoms [50].

### 6.2. Clinical Presentation

Common symptoms of NCWS include diarrhea, bloating, abdominal pain, along with neurological issues like cognitive haze, headache and persistent tiredness [53]. New clinical features of NCWS are emerging, particularly anemia and associated autoimmune conditions [54,55].

### 6.3. Diagnosis

NCGS should be considered when tests for celiac disease and wheat allergy are negative. There are currently no established biomarkers for diagnosing NCGS. Diagnosis relies heavily on linking symptom onset to wheat consumption conclusively. Catassi et al. have developed the so-called “Salerno protocol” for the diagnosis of NCWS, which consists of ruling out CeD and wheat allergy, assessing clinical improvement on a GFD, and a double-blind, placebo-controlled gluten/wheat challenge to confirm the diagnosis [53]. However, many re-challenge studies specific to gluten/wheat are not highly specific [51]. Biomarkers such as zonulin are currently under study, without producing clear results [56,57]. Although not indicated in this type of disorder, some authors found minor changes in the small bowel histology of NCWS, even without significant villous damage [58].

### 6.4. Treatment and Prognosis

Before beginning a gluten-free diet, it is crucial to inform patients suspected of NCGS and their families about potential nutritional deficits linked to the diet [59]. Unlike celiac disease, NCGS is not associated with serious long-term health complications, allowing for a less stringent diet focused primarily on managing symptoms [60]. 

## 7. Eosinophilic Esophagitis (EoE)

Eosinophilic esophagitis (EoE) is a chronic immune-mediated oesophagal disease related to symptoms of oesophagal dysfunction and histologically associated with chronic eosinophil-predominant inflammation with infiltration of the oesophagal mucosa. The disease course of EoE tends to be chronic in most patients, with both clinic and histological alterations being persistent if not properly managed. 

### 7.1. Epidemiology

The overall incidence and prevalence are increasing, even after considering the increase in disease awareness and improvements in diagnostic techniques [61]. A systematic review focusing on the pediatric population has estimated that the occurrence of EoE in children ranges from 0.7 to 10 per 100,000 individuals per year, with prevalence rates varying from 0.2 to 43 per 100,000 [62]. Studies have also revealed that the incidence and prevalence rates were higher in adults than in the pediatric population and in males than in females [61,63].

### 7.2. Pathogenesis

The pathogenesis of EoE is still not fully understood. In genetically predisposed individuals, chronic antigen exposure from food and environmental allergens leads to Th2 cell-mediated inflammation, which is the main driver of the pathophysiology of EoE. This process leads to epithelial changes and the granulocytes’ infiltration of the mucosa, contributing to the processes involved in tissue stiffness [64]. There is now global evidence that food allergens are the most common triggers in EoE. In particular, milk (65–85%), wheat (20–37%), and eggs (10–17%) are the most represented [65]

### 7.3. Clinical Features

Clinical findings of EoE vary depending on the patient’s age of presentation. In adults and young adults, the most common presenting symptoms are associated with oesophagal fibrosis: dysphagia, food impaction, reflux-like symptoms, and chest pain. These symptoms are rarely seen in younger children, but they are commonly seen in patients over the age of 12 years [66]. Younger children or infants present usually with less specific symptoms, such as feeding difficulties, vomiting, and abdominal pain, if untreated EoE progresses to esophageal remodeling, rigidity, and luminal narrowing, associated with a possible severe worsening of all symptoms [67].

### 7.4. Diagnosis

The EoE diagnosis requires the presence of typical symptoms and endoscopic appearance associated with histological findings, and it excludes all the conditions that may lead to symptoms and esophageal eosinophilia.

The histological criteria are satisfied with findings of eosinophil-predominant inflammation on esophageal biopsy. A peak value of ≥15 eosinophils per high power field or 60 eosinophils per square millimeter is often considered diagnostic for EoE [66].

There are some typical macroscopic signs of EoE usually seen at the endoscopy, such as strictures with trachealization of the esophagus, attenuation of the subepithelial vascular pattern, linear furrows, and white spots consisting of eosinophilic microabscesses [66].

### 7.5. Therapy

The current treatment options primarily revolve around the 3Ds: drugs (swallowed topical corticosteroids and proton pump inhibitors), dietary modifications (such as elimination diet of allergens), and endoscopic dilation in cases of advanced disease with esophageal narrowing [68]. Experience is advancing with dupilumab [67]. PPIs are a pharmacological option for the treatment of EoE, and can induce and maintain remission [69]. Most EoE patients respond to swallowed topical glucocorticoids, leading to clinical and histologic improvement [63]. Oral budesonide suspension is favored due to its consistent drug delivery and FDA approval.

#### Dietary Therapy

The most used dietary therapy for EoE is the empiric elimination diet, which is based on avoiding foods commonly related to immediate hypersensitivity. The number of foods eliminated can be increased or decreased progressively [65]. 

Another option is an elimination diet based on SPT/APT, subsequently eliminating foods with positive test results. It leads to improvement in hystologic findings of eosinophilia in a similar percentage of patients as empiric removal of foods [70].

The most effective approach is the elemental diet, in which the patient is placed on an amino acid-based formula that eliminates all potential food allergens [65]. However, this treatment is used rarely because it is very challenging to follow.

### 7.6. Prognosis

The natural history of EoE is not fully understood, but it is recognized as a chronic condition. Untreated, symptoms can persist or occur sporadically, with a high likelihood of recurrence after treatment cessation. While the long-term prognosis is uncertain, EoE does not impact lifespan significantly [71]. Research on EoE’s persistence from childhood to adulthood is limited. Evidence suggests its continuation into adulthood, yet the extent of progressive disease remains unclear [71].

## 8. Summary

Understanding the distinction between wheat and gluten is crucial, as gluten-related conditions and those about wheat should be treated differently. Celiac disease and gluten sensitivity are conditions associated with gluten, necessitating the elimination of all gluten-containing foods from the diet, not just wheat. In contrast, wheat is the culprit in wheat allergies, where simply removing gluten is not sufficient; complete elimination of all wheat and its traces is necessary for safety. Appreciating these distinctions becomes even more important when considering the different pathogenetic mechanisms and, consequently, the clinical outcomes of these conditions. In the case of celiac disease, solely eliminating wheat and not other gluten-containing foods can lead to a progression of symptoms with serious long-term effects due to the persistence of immune-mediated inflammation. Moreover, a gluten-excluding diet that does not eliminate wheat in wheat allergy-related conditions can lead to anaphylaxis or severe acute forms of cellular-mediated reactions, which are life-threatening.

One key difference among these conditions, which share only the term ‘wheat’ but differ in all other aspects, is their varying levels of definition (Table 1). While some conditions like celiac disease and wheat allergy are well established, all the others require further clarification regarding diagnostic and clinical management. In particular, gluten sensitivity remains very poorly understood in all its aspects.

## 9. Conclusions

The term ‘wheat-related diseases’ encompasses conditions that share only the commonality of ‘wheat’ yet diverge significantly in pathogenesis, clinical manifestation, diagnosis, and treatment. Pediatricians must familiarize themselves with these diverse forms to optimize management and avoid misdiagnosis or incorrect management, which could be risky. This is especially important considering that these conditions strongly impact the quality of life, affecting both health and social life, and are seeing a rise in prevalence.

## Figures and Tables

**Table 1:** Wheat-related disorders overview.

	Celiac Disease	Non Celiac-WS	IGE-Wheat Allergy	Non-IGE-Wheat Allergy	Eosinophilic Esophagitis
Causative foods	Gluten containing food	Wheat	Wheat	Milk 40–50% (less commonly soy, egg, wheat, corn)	Wheat, milk, egg
Pathogenesis	Autoimmune response against gluten peptides	Unknown	Wheat specific IgE antibodies	Cellular immunity responses, with T-cells infiltration of the intestinal mucosa	Chronic immune reaction triggered by food antigens; Th2 cell-mediated inflammation and eosinophils infiltration
Symptoms and sign	Symptoms and sign of malabsorption, GI symptoms	IBS-like, headache and fatigue	Acute: GI symptoms, anaphylaxis	Acute or chronic GI symptoms; vary by specific condition	Dysphagia, food impaction, reflux like symptoms, chest pain
Diagnosis	Serologic test, EGDS	Exclusion of other wheat-related disease, confirmation with gluten challenge		Clinical history, typical symptoms improvement upon food withdrawal and exclusion of other etiologies	Endoscopic appearance histological findings of esophageal eosinophilia; exclusion of other conditions causing esophageal eosinophilia
Treatment	GFD	GFD, low FODMAP diet		Removal of trigger foods from the patient’s diet and maternal diet if breastfeeded	Swallowed topical corticosteroids, proton pump inhibitors, dietary modifications, endoscopic dilation

EGDS: Esophago-Gastro-Duodeno-Scopy; FODMAP: Fermentable, Oligo-, Di-, Mono-saccharides And Polyols; GFD: Gluten Free Diet; GI: Gastro-Intestinal; IBS: Irritable Bowel Syndrome.

## Data Availability

Not applicable.
